# The Anticancer Activities of Natural Terpenoids That Inhibit Both Melanoma and Non-Melanoma Skin Cancers

**DOI:** 10.3390/ijms25084423

**Published:** 2024-04-17

**Authors:** Ye Eun Yoon, Young Jae Jung, Sung-Joon Lee

**Affiliations:** 1 Department of Biotechnology, Graduate School of Life Sciences & Biotechnology, College of Life Sciences and Biotechnology, Korea University, Seoul 02855, Republic of Korea; lyjung51@korea.ac.kr; 2 Department of Food Bioscience and Technology, College of Life Sciences and Biotechnology, Korea University, Seoul 02855, Republic of Korea; 3 Interdisciplinary Program in Precision Public Health, BK21 Four Institute of Precision Public Health, Korea University, Seoul 02846, Republic of Korea

**Keywords:** skin cancer, melanoma, non-melanoma skin cancer, natural terpenoids, phytochemicals

## Abstract

The prevalence of two major types of skin cancer, melanoma and non-melanoma skin cancer, has been increasing worldwide. Skin cancer incidence is estimated to rise continuously over the next 20 years due to ozone depletion and an increased life expectancy. Chemotherapeutic agents could affect healthy cells, and thus may be toxic to them and cause numerous side effects or drug resistance. Phytochemicals that are naturally occurring in fruits, plants, and herbs are known to possess various bioactive properties, including anticancer properties. Although the effects of phytochemicals are relatively milder than chemotherapeutic agents, the long-term intake of phytochemicals may be effective and safe in preventing tumor development in humans. Diverse phytochemicals have shown anti-tumorigenic activities for either melanoma or non-melanoma skin cancer. In this review, we focused on summarizing recent research findings of the natural and dietary terpenoids (eucalyptol, eugenol, geraniol, linalool, and ursolic acid) that have anticancer activities for both melanoma and non-melanoma skin cancers. These terpenoids may be helpful to protect skin collectively to prevent tumorigenesis of both melanoma and nonmelanoma skin cancers.

## 1. Introduction

Skin, constituting approximately 16% of body mass, serves as a physiochemical barrier against daily attacks such as scratches, wounds, and environmental stressors, like chemicals, pathogens, and ultraviolet (UV) radiation [1,2]. As skin occupies the largest area of the body and each layer of skin has its distinctive function, maintaining skin health is crucial. However, the skin cancer incidence is dramatically increasing due to increased UV exposure, environmental factors, hereditary risk elements, and enhanced surveillance leading to earlier recognition, and has become a problem worldwide [3].

Skin cancers are mainly divided into the following two types: melanoma and non-melanoma skin cancer (NMSC). Melanoma is a cancer that arises from the melanocytes in the epidermis. Melanoma, particularly cutaneous malignant melanoma (CMM), is renowned as the most aggressive form of skin cancer, distinguished by its likelihood of metastasis and elevated mortality rates [4]. CMM is frequently detected on the lower legs of women and the trunk of men, although it commonly occurs on the head and neck [5]. It appears most commonly as a pigmented lesion that varies from dark brown to blue–black with an irregular asymmetrical shape [6]. NMSC is derived from epidermal keratinocytes, occur on UV-exposed areas, and are often asymptomatic. NMSC is divided into the following two types: basal cell carcinoma (BCC) and squamous cell carcinoma (SCC). BCCs and SCCs have different morphology, incident rates, and etiological differences. BCCs most commonly appear as pearly white, dome-shaped papules, often with telangiectasia; SCCs most commonly appear as smooth or hyperkeratotic papules with a central ulceration [7]. They have a much lower mortality than CMM because they tend to remain confined to their primary site of disease.

BCCs, constituting 80–85% of all NMSCs, rarely metastasize to other organs [8]. Despite its low mortality rate, this form of malignancy significantly impacts morbidity and places a substantial burden on healthcare systems globally [9,10]. SCCs, comprising 15–29% of all NMSCs, presents a higher likelihood of tissue and bone invasion, potentially leading to fatal outcomes [8,9]. Reports from both the U.S.A. and Europe indicate a progressive and concerning rise in NMSC incidence [11]. While historically prevalent among Caucasians, recent years have witnessed an alarming increase in NMSC cases among the Hispanic and Asian populations, underscoring a concerning trend in these demographics [12].

Skin cancers, including both NMSC and malignant melanoma, originate from a complex interplay of genetic predisposition and environmental factors, primarily UV radiation exposure [1,2]. UV radiation damages the DNA of skin cells, leading to the accumulation of genetic mutations and alterations in key regulatory genes such as TP53, CDKN2A, and BRAF [13]. These mutations disrupt the cellular signaling pathways involved in cell cycle control, DNA repair, and apoptosis, thereby promoting uncontrolled cell proliferation and survival. In NMSC, prolonged UV exposure predominantly triggers the development of BCC and SCC, characterized by abnormal growths in the basal and squamous layers of the epidermis, respectively [14]. Conversely, malignant melanoma arises from melanocytes, pigment-producing cells scattered throughout the epidermis, dermis, and mucous membranes, where mutations in genes, such as BRAF and NRAS, drive melanocyte transformation and metastasis [15]. As skin cancers progress, they can invade nearby tissues, spread to nearby lymph nodes, and metastasize to distant organs, presenting substantial health hazards.

Skin cancer is commonly addressed through various treatment methods such as surgical excision, radiation therapy, chemotherapy, and cryosurgery. Topical medications, like 5-fluorouracil (5-FU) and imiquimod, are employed in chemotherapy regimens for superficial BCC and SCC in situ, while imiquimod is only licensed for topical therapy of CMM [16]. However, the main issues with chemotherapeutic medicines are the severe side effects and the development of multi-drug resistance. Since chemotherapy works by targeting active cells, both cancer cells and healthy cells that are growing and going through the normal cell cycle gets damaged. Patients commonly experience fatigue, loss of appetite, hair loss, burning pain from nerve damage, and blood disorders due to a low number of blood cells. Despite the utilization of chemotherapy, cancer cells may acquire resistance to treatment through mechanisms like drug efflux systems, amplification of drug targets, and alterations in drug kinetics. While symptomatic management can alleviate chemotherapy side effects, secondary treatments can sometimes be excessively toxic, which certain cancer patients find unacceptable. The increasing incidence of skin cancer and the negative effects linked with existing treatment methods emphasize the importance of exploring a range of alternative treatment options.

Phytochemicals, derived from plant extracts, have potential anticancer properties and serve as lead compounds for novel drug development and terpenoids are one such example. Terpenoids are synthesized through the mevalonate pathway in plants and some microorganisms, while in animals, including humans, they are synthesized through the methylerythritol phosphate pathway [17]. They are frequently used in traditional medicines in the form of teas or crude extracts. Terpenoids can be classified into subclasses according to the number of isoprene units, such as hemiterpenoids (C5), monoterpenoids (C10), sesquiterpenoids (C15), diterpenoids (C20), triterpenoids (C30), tetraterpenoids (C40), including carotenoids, and polyterpenoids (C5)n. Many terpenoids are reported to have an anti-cancerous effect against breast, gastric, and colorectal cancers [18,19,20,21]. Some terpenoids are already being used for treating cancers. Artemisinin, which belongs to the sesquiterpene group, is a well-known antimalarial drug that is also reported to exert anti-tumor effects in vitro and in vivo with minor side effects [22,23]. For instance, a semisynthetic derivative of artemisinin, artesunate, is used to treat melanoma and various cancers, including colon, lung, prostate, breast, and ovarian cancers [24].

Currently, numerous studies have reviewed the effect of terpenes with the focus on either malignant melanoma or SCC. Kłos et al. summarized 18 plant-derived terpenoids that have anti-melanoma activity [25]. In another review, Wróblewska-Łuczka et al. described anti-melanoma activities of 15 different terpenes with a focus on how these terpenes can be used as adjuvant therapy in melanoma treatment such as chemotherapy and immunotherapy [26]. Some lesser-known triterpenoids that have anti-melanoma effect have been described previously [27]. Along with other natural compounds, some terpenoids, including ingenol mebutate, glycyrrhizic acid, and botulin, are known to prevent NMSC [28].

In this review, we selected terpenoids that act on preventing both melanoma and non-melanoma skin cancers and aimed to summarize the underlying mechanisms. Although considered not common, combined tumors of malignant melanoma and non-melanoma skin cancer, termed “squamomelanocytic” tumors have been reported [29,30]. Currently, squamomelanocytic tumors are treated as malignant melanoma, but the prognosis remains uncertain. The selected terpenoids in this review may be useful to prevent both melanoma and non-melanoma skin cancers or to prevent squamomelanocytic tumors. Thus, here in this paper, we aimed to summarize five natural terpenoids, eucalyptol, eugenol, geraniol, linalool, and ursolic acid, that inhibit both NMSC and malignant melanoma.

## 2. Anticancer Activities of Natural Terpenoids

This section deals with the activity of the terpenoids against melanoma and NMSC. Table 1 shows the source of each terpenoid and the mechanism of the anti-melanoma and anti-NMSC effects induced by each phytochemical. 

### 2.1. Eucalyptol

Eucalyptol, 1,8-cineole, is a natural monoterpenoid compound that is abundant in plants, such as *Rosmarinus officinalis*, *Eucalyptus globulus*, and *Salvia fruticosa* [31,50,51], which is known to exhibit anti-inflammatory [52,53] and anti-oxidative effects [54]. UVB irradiation induces cyclooxygenase-2 (COX-2) and abnormal expression of COX-2 is associated with various types of cancer including skin cancer [55,56]. In NMSC, Lee et al. reported that 1,8-cineole inhibited UVB-induced COX-2 protein and mRNA expression, and prostaglandin E2 (PGE_2_) generation in human keratinocytes, HaCaT, by targeting the aryl hydrocarbon receptor (AhR) [31]. AhR is a ligand-activated transcription factor where environmental pollutants such as benzo[α]pyrene can act as an agonist and cause inflammation and carcinogenesis [57,58]. Eucalyptol acted as an AhR inhibitor and also delayed tumor incidence and the tumor numbers in the UVB-induced SKH-1 mice, while inhibiting COX-2 expression when applied topically [31].

Eucalyptol-suppressed NMSC proliferation via G2/M cell cycle arrest, upregulating P53 signaling pathway and inducing apoptosis assessed by modulating apoptotic markers, such as Bax/Bcl-2, cytochrome c, caspase-3 and caspase-9 in the human SCC cell line, A431 cells [32]. Rahaman et al. reported the effect of eucalyptol on metastasis for both melanoma and SCC in vitro and in vivo [33]. In vitro, eucalyptol significantly decreased migration and invasion by inhibiting the PI3K/Akt/mTOR pathway in A431 human SCC cells, A375 human melanoma cells, and B16F10 mouse melanoma cells, respectively. The PI3K/Akt/mTOR pathway is a signaling pathway highly associated with cancer progression. Eucalyptol also reversed the epithelial to mesenchymal transition (EMT) by reducing the expression of the mesenchymal markers, vimentin, snail, slug, twist, MMP2, MMP9, n-cadherin, and inducing the expression of the epithelial marker, E-cadherin, in A431, A375, and B16F10. The anti-metastatic activity of eucalyptol was evaluated in vivo by injecting B16F10 melanoma cells into mice via the tail vein. Administration of eucalyptol inhibited the metastasis of B16F10 cells to the lung tissue with a reduction in vimentin expression [33].

**Figure 1:**
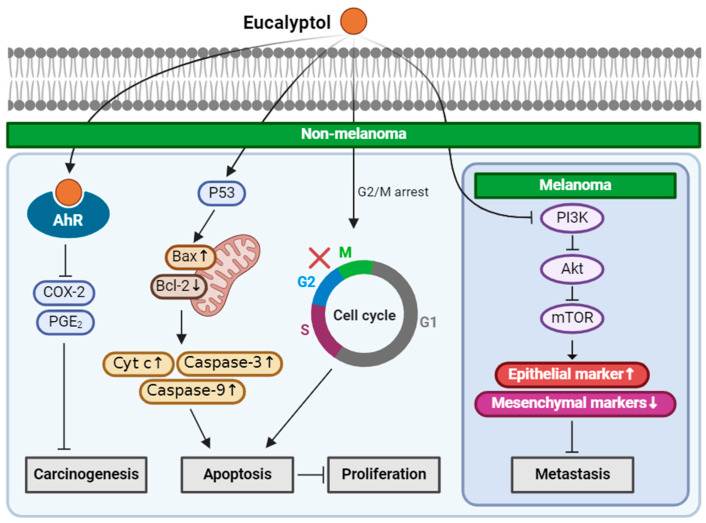
Schematic of the mechanisms involved in the anti-skin-cancer effects of eucalyptol. In NMSC, eucalyptol functions as an AhR agonist and suppresses UVB-induced COX-2 and PGE_2_, and inhibits carcinogenesis. By upregulating P53, eucalyptol increases apoptotic markers, such as Bax, cytochrome c, caspase-3, caspase-9, and decreases Bcl-2 that leads to apoptosis and blocks cell proliferation. G2/M cell cycle by eugenol also leads to apoptosis. Inhibition of the PI3K/Akt/mTOR pathway reduces the metastasis of NMSC, and it is also involved in the anti-metastasis of melanoma, increases epithelial markers, and decreases mesenchymal markers. ↓: decrease in expression; ↑: increase in expression; × (red): arrest in cell cycle.

These results suggest that eucalyptol may be a promising treatment in inhibiting the spread of NMSC and melanoma. In NMSC, eucalyptol inhibits UVB-induced COX-2 expression via AhR, thereby inducing G2/M cell cycle arrest, which then leads to apoptosis. Metastasis is also inhibited by targeting the PI3K/Akt/mTOR pathway. The PI3K/Akt/mTOR signaling axis is also the target for inhibiting the metastasis of melanoma (Figure 1). Thus, eucalyptol can be a promising natural compound that can be used to treat skin cancers.

### 2.2. Eugenol

Eugenol, 4-allyl (-2-methoxyphenol), is a monoterpenoid that can be found naturally in spices and herbs, such as nutmeg, cinnamon, cloves, and basil [59,60]. It is used as a flavoring agent in food products, such as teas and cakes, and cosmetic products like perfumes. The various antioxidant, antiviral, and anti-inflammatory effects of eugenol have been described [61]. The anticancer effect of eugenol-rich agents, such as betel leaf extract [62] and clocimum oil [63], was reported but the molecular mechanisms by which eugenol alone exerts its anticancer effect are largely unknown.

Kaur et al. investigated the protective effect of eugenol against NMSC in Swiss albino mice. Tumors were first initiated in the mice by applying a carcinogen, 7, 12-dimethylbenz[α]anthracene (DMBA), on the skin. A single application of DMBA can achieve tumor initiation via DNA mutation. The skin tumors were then promoted by applying 12-O-tetradecanoylphorbol-13-acetate (TPA) twice weekly for 28 weeks. TPA stimulates cell proliferation by activating protein kinase C (PKC). Pretreatment with eugenol delayed the tumor development and a smaller number of tumors formed compared to the DMBA- and TPA-treated control group. Immunohistochemistry of the proliferation marker, proliferating cell nuclear antigen (PCNA), and TUNEL staining revealed the anti-proliferation and pro-apoptotic effects of eugenol. The apoptosis was stimulated by the increased protein expression of the DNA damage biomarker, P53 and P21^WAF1^, after eugenol pretreatment. Eugenol markedly repressed the inflammation biomarkers, iNOS and COX-2, in response to attenuated levels of phospho-IkBα and the suppressed accumulation of NF-κB in the nucleus. TPA-induced proinflammatory cytokines, such as IL-6, TNF-α, and PGE_2_, that are known to increase vascular permeability, epidermal hyperplasia, and inflammatory cell infiltration [64], was also reduced via eugenol treatment [34].

The anticarcinogenic effect of eugenol was also studied in the NMSC model of DMBA/croton oil-induced skin carcinogenesis in Swiss albino mice [35]. Croton oil is a poisonous viscous liquid obtained from the seeds of *Croton tiglium.* The tumor was induced by topical application of DMBA and croton oil. Eugenol was orally administered 15 days prior to DMBA and croton treatment. Notably, eugenol treatment reduced the tumor incidence and sizes of the skin tumors with an increase in the overall survival rate of the mice. Eugenol reduced cell proliferation by downregulating mRNA and protein expression of the two oncogenes, c-Myc and H-ras. Eugenol treatment induced apoptosis in the skin lesions of the mice by downregulating the antiapoptotic gene, Bcl2, and upregulating the proapoptotic genes, Bax, p53, and active caspase-3 [35].

Eugenol was also found to be a potent inhibitor of melanoma cell proliferation. Eugenol treatment delayed the tumor growth by 19% and decreased the size of the tumors by 62% in the B16F10 xenograft mice [36]. Moreover, 50% of mice in the control group showed metastasis, while none in the eugenol-treated group showed any signs of invasion or metastasis. TUNEL assay of the tumor sections showed that eugenol induces apoptosis in melanoma tumors. The mechanism of anti-proliferation was evaluated using a human malignant melanoma cell line, WM1205Lu, and the results showed that eugenol causes a cell cycle arrest in the S phase, triggering apoptosis [36]. The E2F proteins, a group of transcription factors, play a crucial role in controlling the progression of the cell cycle [65]. Dysregulated transcriptional activity of E2F family within the melanoma cells drives ongoing proliferation, with E3F2 and E2F4 being especially prominent in actively dividing melanoma cells [66]. Eugenol treatment inhibited E2F1 transcriptional activity in WM1205Lu, suggesting a mechanism for its antiproliferative effects [36]. The mechanism of anti-metastasis still needs further investigations.

**Figure 2:**
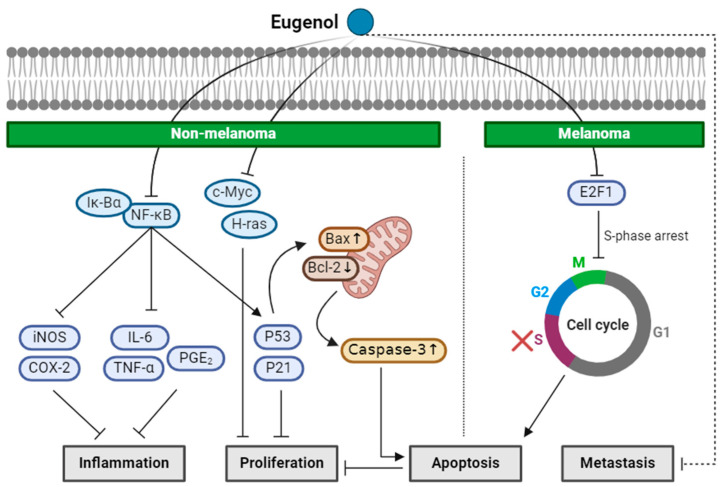
Schematic of the mechanisms involved in the anti-skin-cancer effects of eugenol. In NMSC, eugenol inhibits NF-κB, repressing inflammation markers iNOS, COX-2, IL-6, TNF-α, and PGE_2_. Inhibition of NF-κB induces P53 and P21^WAF1^, which leads to an increase in the Bax/Bcl ratio and active caspase-3, inducing apoptosis. In melanoma, eugenol suppresses E2F1 expression that causes the S-phase cell cycle arrest, leading to apoptosis. Eugenol is also able to inhibit metastasis, but the underlying mechanisms have yet to be elucidated. ↓: decrease in expression; ↑: increase in expression; × (red): arrest in cell cycle.

These findings collectively suggest that eugenol can effectively protect against chemically induced NMSC and melanoma. Eugenol exhibited protective effects against tumor initiation and promotion in vivo by promoting apoptosis and attenuating proliferation via the upregulation of P53 and P21^WAF1^. It also reduced inflammation by inhibiting NF-κB. Additionally, in another vivo model, eugenol decreased tumor incidence and size while increasing overall survival by downregulating oncogenes like c-Myc and H-ras and inducing apoptosis through Bcl2 downregulation and Bax upregulation. In melanoma, eugenol inhibited the tumor growth and metastasis in xenograft mice models and induced apoptosis by triggering an S phase cell cycle arrest and inhibiting E2F1 transcriptional activity (Figure 2). However, further mechanistic investigations are warranted, especially on how eugenol inhibits metastasis.

### 2.3. Geraniol

Geraniol, an acyclic monoterpene, is a constituent of essential oils from aromatic plants, such as *Cinnamomum tenuipilum* and *Phyla scaberrima* [67]. It also occurs in citrus fruits like lemons and grapefruits [68]. Geraniol is recognized for its antimicrobial, antidiabetic, and antiarrhythmic effects [69,70,71]. Moreover, it demonstrates antiproliferative properties against oral, colon, lung, liver cancer [67]. Yet, there is a lack of information regarding its effects on human skin cancer.

Fatima et al. investigated the antiproliferative potential of geraniol in NMSC [37]. Geraniol inhibited the proliferation of A431 cells by suppressing the activity of lipoxygenase-5 (LOX-5) and hyaluronidase, and then induced apoptosis by causing a G0/G1 cell cycle arrest [37]. LOX-5 is an anti-inflammatory molecular target for cancer drug development. When LOX-5 is abnormally expressed in human cancers, such as pancreas, prostate, and colon cancer, it synthesizes leukotrienes and promotes proliferation, apoptotic resistance, invasion, migration, and angiogenesis in cancer cells. [72]. Hyaluronidase is a glycosylated protein that cleaves hyaluronan in the extracellular matrix. The fragmentation of hyaluronan enhances adhesion and elasticity of the cells and thus is related to invasion, angiogenesis, and metastasis [73]. Same as LOX-5, the clinical report indicates that hyaluronidase is overexpressed in cancer cells [74]. Thus, the suppression of LOX-5 and hyaluronidase in A431 by geraniol can help inhibit proliferation and metastasis.

Geraniol exhibited anti-NMSC properties and pro-apoptotic effects in in vivo experiments involving Swiss albino mice subjected to skin tumorigenesis induced by DMBA and TPA [38]. The topical application of geraniol 30 min before TPA treatment significantly mitigated TPA-induced skin edema, hyperplasia, COX-2 induction, and oxidative stress response [38]. Moreover, geraniol inhibited tumor formation and reduced the number of tumors, while also extending the latency period from 4 weeks in the control group to 10 weeks in the geraniol-pretreated group. These effects were achieved by suppressing the Ras/Raf/ERK1/2 signaling pathway in skin tumors and inducing a pro-apoptotic state with a decreased Bcl-2/Bax ratio [38]. Khan et al. also demonstrated the protective effect of geraniol against TPA-induced oxidative stress [75]. Topical pre-treatment of geraniol 30 min prior to TPA administration significantly inhibited TPA-induced lipid peroxidation, inflammatory responses, proinflammatory cytokine release, and upregulated glutathione content. These results suggest that geraniol shows anti-proliferation and anti-tumor effects in vitro and in vivo, by suppressing LOX-5 and Ras/Raf/ERK1/2 pathway.

Geraniol also demonstrated potent anticancer properties against malignant melanoma. Yu et al. observed a dose-dependent effect of geraniol on the growth of B16F10 melanoma in mice [39]. When administered through the diet 14 days prior and for 21 days following tumor transplantation, geraniol significantly impeded tumor growth. This effect may be attributed to geraniol’s ability to inhibit the activity of 3-hydroxy-3-methylglutaryl coenzyme A (HMG-CoA) reductase, thereby hindering the proliferation of melanoma cells [39]. Farnesyl pyrophosphate, the intermediate product of the mevalonate pathway, is critical for the posttranslational modification of proteins, such as P21, prelamin A and lamin B, which play an important role in cell proliferation. Therefore, an HMG-CoA reductase inhibitor like lovastatin can inhibit nuclear lamins. However, lovastatin is difficult to use since the inhibitory dose is toxic to normal cells. Dietary geraniol suppresses the activity of HMG-CoA reductase. Inhibition of the isoprenylation of nuclear lamins is known to trigger apoptosis [76]. In-depth future investigations are needed to reveal the mechanisms underlying the apoptosis.

**Figure 3:**
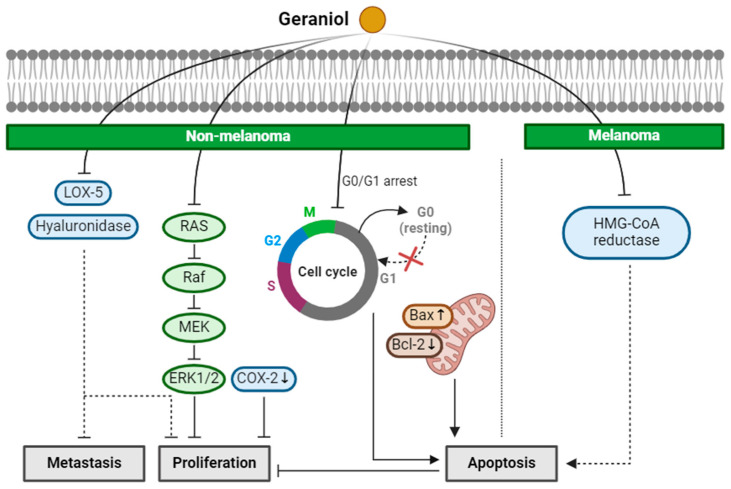
Schematic of the mechanisms involved in the anti-skin-cancer effects of geraniol. In NMSC, geraniol inhibits LOX-5 and hyaluronidase activity, which reduces metastasis and proliferation. Decreased COX-2 expression and inhibition of the Ras/Raf/ERK1/2 pathway is involved in the anti-proliferation of NMSC by geraniol. Geraniol also induces G0/G1 cell cycle arrest in NMSC that leads to apoptosis. In melanoma, HMG-CoA reductase inhibition is found to cause apoptosis. ↓: decrease in expression; ↑: increase in expression; × (red): arrest in cell cycle.

Collectively, these findings show that geraniol has the potential of inhibiting skin cancers. In NMSC, geraniol inhibited proliferation and metastasis through the suppression of LOX-5 and hyaluronidase activities. Eugenol also induced apoptosis by arresting the cell cycle at the G0/G1 phase. In vivo, the proliferation of tumors was reduced by suppressing the Ras/Raf/ERK1/2 pathway and decreasing the Bcl-2/Bax ratio by geraniol treatment. In melanoma, geraniol demonstrated a proapoptotic effect by inhibiting HMG-CoA reductase activity, suggesting potential as an anti-tumor agent. Yet, further investigation is needed to understand the specific mechanisms of apoptosis induction in melanoma (Figure 3).

### 2.4. Linalool

Linalool (2,6-dimethyl-2,7-octadien-6-ol), an acyclic monoterpene that naturally occurs in different aromatic plants, such as *Cinnamomum tenuipilum*, *Coriandrum sativum*, and *Lavandula angustifolia* [77], was evaluated by Gunaseelan et al. for its ability to suppress NMSC in mice exposed to chronic UVB radiation [40]. The study found that pre-treatment with linalool before every UVB exposure significantly reduced the expression of proliferation markers, such as NF-κB, TNF-α, IL-6, COX-2, VEGF, TGF-β1, Bcl-2, which were elevated by chronic UVB exposure. This decrease in marker expression correlated with a reduction in tumor incidence in the mice’s skin. Histopathological examinations confirmed that dysplasia and SCC development, induced by chronic UV-B exposure, were prevented by both the topical application and intraperitoneal injection of linalool. Thus, linalool may serve as a photochemopreventive agent against UVB radiation-induced skin carcinogenesis [40].

Furthermore, the antiproliferative effect of linalool on the RPMI 7932 human melanoma cells was investigated in vitro. Linalool inhibited the growth of melanoma cells and induced morphological changes, such as chromatin rearrangements, condensation, nuclear fragmentation and formation of apoptotic bodies [41]. However, this antiproliferative effect and associated morphological changes were not observed in the NCTC 2544 normal keratinocytes, suggesting that keratinocytes are more resistant to linalool than melanoma cells [41]. The study also noted a slight increase in nuclear caspase-3 in RPMI 7932 cells treated with linalool, indicating intracellular translocation of the active caspases critical for the apoptotic process [41,78]. Although further research is needed to elucidate the molecular mechanism behind linalool’s antiproliferative activity on melanoma cells, this study suggests that linalool could potentially be utilized in the development of therapeutic agents for melanoma.

Anticancer potential of linalool has been investigated in mouse B16F10 as well. Linalool significantly inhibited angiogenesis and metastasis, reducing CoCl_2_-induced HIF-1α expression and reducing VEGF secretion from the cell [42]. Linalool also reduced the mesenchymal markers, vimentin, MMP2 and MMP9, and upregulated the epithelial marker, E-cadherin. However, a higher concentration of linalool significantly increased angiogenesis and metastasis, showing bimodal activity depending on concentrations [42].

**Figure 4:**
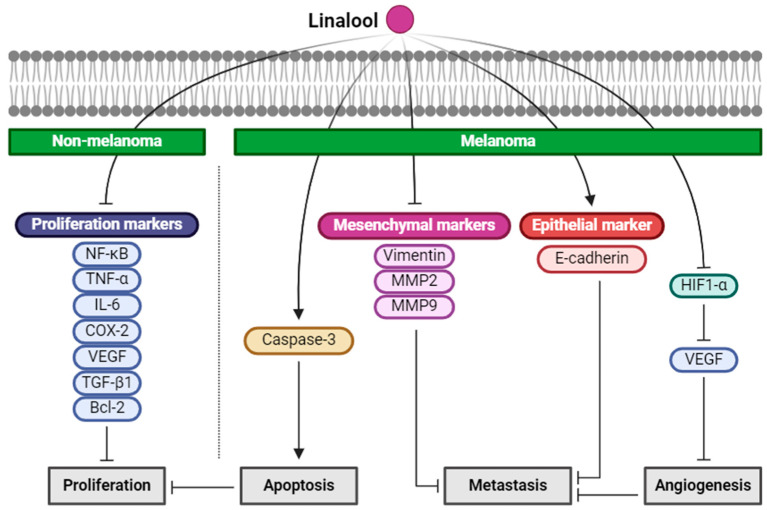
Schematic of the mechanisms involved in the anti-skin-cancer effects of linalool. Linalool suppresses the proliferation markers, including NF-κB, TNF-α, IL-6, COX-2, VEGF, TGF-β1, Bcl-2, inhibiting the proliferation of NMSC. In melanoma, linalool increases caspase-3, leading to apoptosis and decreasing proliferation. Linalool also decreases the mesenchymal markers, vimentin, MMP2, and MMP9, and increases the epithelial marker, E-cadherin, inhibiting metastasis of melanoma.

Taken together, linalool showed an anticancer effect on both NMSC and melanoma. Linalool reduced the expression of proliferation markers that were elevated by UVB in the NMSC animal model, decreasing tumor incidence. In human melanoma cells, the results revealed linalool’s antiproliferative effects, inducing apoptotic changes without affecting the normal keratinocytes, suggesting its specificity towards cancer cells. Additionally, linalool inhibited angiogenesis and metastasis in the mouse melanoma cells by reducing HIF-1α expression and VEGF secretion, and by modulating the markers of epithelial–mesenchymal transition (Figure 4). While a concentration-dependent bimodal activity of linalool warrants further investigation for therapeutic development, the results of this study sheds light on the potential of linalool as anti-skin-cancer agent.

### 2.5. Ursolic Acid

Ursolic acid (UA) is a natural triterpenoid that is enriched in fruits and plants, such as blueberry, cranberry, apple, and *Salvia fruticosa* [79,80]. Kowalczyk et al. investigated, in vitro, the potential NMSC-preventive properties of UA to define the mechanisms by which these compounds may inhibit murine skin carcinogenesis [43]. The following three types of cell lines representing different stages of cancer were used: 3PC cell line representing non-tumorigenic cells, the papilloma-derived MT1/2 cell line, and the squamous cell carcinoma-derived Ca3/7 cell line. UA exhibited scavenging activity against the reactive oxygen species (ROS) and prevented DNA from hydrogen peroxide-induced damage in Ca3/7 cells. Furthermore, UA inhibited the growth of all three cell lines by enhancing the activities of caspase-3 and -7, ultimately inducing apoptosis [43]. Acting as a master regulator of the anti-oxidative stress response, nuclear factor E2-related factor 2 (Nrf2) exhibits anticarcinogenic activity against the skin tumor development triggered by chemicals and UV radiation [81]. UA was found to restore the expression of the epigenetically silenced *Nrf2* gene in mouse JB6 P+ epidermal cells by demethylating CpG islands within the *Nrf2* promoter, resulting in increased mRNA and protein expression of Nrf2 [44]. As a result, Nrf2’s downstream detoxifying/antioxidant related genes and protein levels of heme oxygenase-1 (HO-1), NAD(P)H:quinone oxidoreductase 1 (NQO1), and uridine 5′-diphospho-glucuronosyltransferase (UGT) increased [44]. This subsequently inhibited TPA-induced neoplastic transformation by 30% in JB6 P+ cells.

The efficacy of UA was further evaluated in vivo on UVB-induced NMSC in SKH-1 hairless mice [45]. Samples from both epidermis and tumors of SKH-1 mice treated topically with UA were collected at the early, transitional, and late stages. Morphologically, UA reduced both tumor volume and number, indicating its potent chemopreventive effects [45]. RNA sequencing analysis revealed significant upregulation of *Nrf2* and *Nqo1* by UA during the early stage of UVB-induced carcinogenesis. Consistent with this finding, methylation analysis demonstrated hypomethylation of these genes at their CpG sites [45].

Additionally, the effects of UA were assessed in B16F10 mouse melanoma cells. Nontoxic concentrations of UA induced apoptotic bodies and DNA fragmentation, increasing the apoptotic genes, *p53* and *caspase-3,* while downregulating the anti-apoptotic gene *Bcl-2* [46]. NF-κB, known for its role in immune and inflammatory responses [82], acts as an anti-apoptotic factor. UA treatment significantly inhibited NF-κBp65, NF-κBp50, NF-κBc-Rel, c-FOS, ATF-2, and CREB-1, along with downregulation of inflammatory genes, such as *Tnfa*, *Il1b*, *Il6* and granulocyte-macrophage colony-stimulating factor (GM-CSF) [46]. These findings suggest that UA induces apoptosis by inhibiting the NF-κB-mediated anti-apoptotic pathway and activating the p53-mediated pro-apoptotic pathways.

Furthermore, the cytotoxic effects of UA were explored in various human melanoma cells, including MM200, Mel-RM, Me4405, and A375. UA demonstrated antiproliferative effects on these cell lines by inducing both early and late apoptosis, accompanied by activation of caspase-3 and -8 [47]. UA also induced apoptosis through the mitochondrial intrinsic pathway, as evidenced by the transmembrane potential collapse and caspase-3 activation in M4Beu human melanoma cells. Additionally, UA altered the Bax–Bcl-2 balance, increasing Bax expression and decreasing Bcl-2 expression [48]. In the SK-MEL-2 human melanoma cell line, UA exhibited intense antiproliferative activity by inducing cell cycle arrest in the S phase [49].

**Figure 5:**
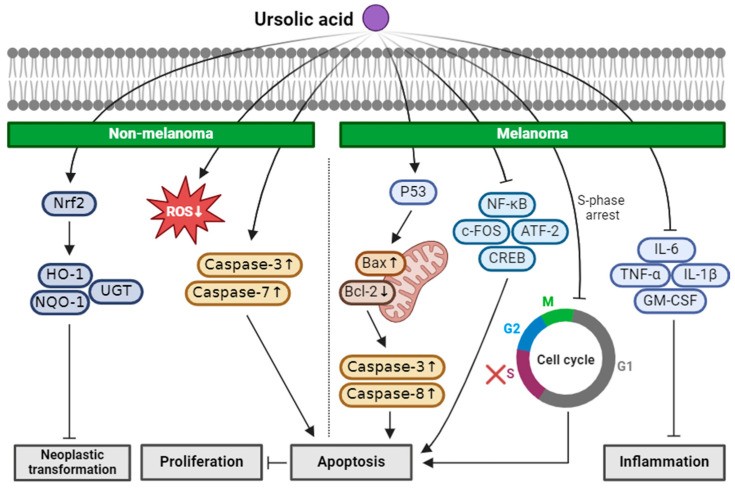
Schematic of the mechanisms involved in the anti-skin-cancer effects of ursolic acid. UA restores the expression of Nrf2 in NMSC and this upregulates HO-1, NQO-1, and UGT, preventing neoplastic transformation. UA also functions as an ROS scavenger, preventing DNA damage and inducing apoptosis by increasing active caspase-3 and caspase-7. In melanoma, UA induces apoptosis by increasing P53, Bax, caspase-3, caspase-8, and decreasing Bcl-2. UA downregulates NF-κB, c-FOS, ATF-2, and CREB, and induces the S-phase cell cycle arrest, which also leads to apoptosis. Inflammation is reduced by UA with decreased inflammatory markers, including IL-6, TNF-α, IL-1β, and granulocyte-macrophage colony-stimulating factor (GM-CSF). ↓: decrease in expression; ↑: increase in expression; × (red): arrest in cell cycle.

In summary, UA demonstrated promising preventive and therapeutic properties against NMSC and melanoma. In vitro studies revealed UA’s ability to scavenge ROS, prevent DNA damage, and induce apoptosis in NMSC cell lines through the activation of caspases and restoration of the Nrf2 pathway. In vivo experiments of NMSC demonstrated UA’s reduced tumor volume and number, along with the upregulation of the Nrf2 and Nqo1 genes. Furthermore, UA induced apoptosis in melanoma cells, which was mediated by the modulation of the apoptotic and inflammatory pathways, including the inhibition of NF-κB, the induction of S-phase cell cycle arrest, and the alteration of the Bax/Bcl-2 balance, showing its potential as a chemopreventive and therapeutic agent against skin cancer (Figure 5). UA shows promise as a potential compound for treating or preventing skin cancer and could emerge as a promising new anticancer agent. Expansion of the abbreviations used in this paper can be found in Table S1. 

## 3. Conclusions

Despite significant progress in the field of cancer diagnosis and chemotherapy, skin cancer still remains one of the common cancers that causes significant mortality. In the realm of cancer treatment, innovative methods often encounter obstacles, primarily due to frequent genetic changes and mutations found within cancer cell DNA. Given the substantial side effects associated with traditional chemotherapy, there is an urgent need for improved and more efficient therapies, especially for advanced stage skin cancers that have spread to other parts of the body. Consequently, there is a growing enthusiasm for the development of treatments involving natural compounds. Numerous studies carried out by several investigators in vitro and in vivo demonstrated the anticancer activities of terpenoid phytochemicals. The major mechanisms of these terpenoids in the chemoprevention of melanoma and non-melanoma include inhibiting proliferation, inflammation, metastasis, and promoting apoptosis. However, adverse effects of such natural compounds must be taken to account before choosing them as treatment options.

An increasing number of cases require closer attention to the potential toxicity induced by natural compounds. The toxicity of these compounds largely depends on their chemical structure, treatment concentration, route of exposure, and physiochemical properties. Often, natural compounds are applied directly to the problem areas as oils or creams, and based upon their lipophilicity, they may be readily absorbed by the skin. Applying excessive amounts of a high concentration to a large area of vulnerable skin could result in systemic absorption and increase serious side effects such as convulsion. Oral consumption of natural products can also pose problems. A meta-analysis collected 97 cases of herbal-induced toxicity in Korea, revealing that both single and multiple herbal preparations could induce hepatocellular toxicity [83]. Numerous studies have summarized some common terpenes, such as camphor and limonene, possibly causing liver injury by producing ROS and impairing antioxidant defenses [84]. Modulation of cytochrome P450 is another hepatotoxic mechanism concurrently observed [85]. These findings from the use of natural compounds in treating cancer indicate that safety measures should be implemented to prevent liver or other potential damages caused by these compounds.

On the other hand, natural compounds can also have a synergistic effect when used with another natural compound or other therapies. To the best of our knowledge, the synergistic effect of the terpenoids, discussed in this review, on skin cancer has never been explored. However, research has shown that other terpenes do exert synergistic effects when used concomitantly with targeted therapies, such as BRAF and MEK inhibitors. For instance, β-caryophyllene, a terpene found in many essential oils, enhanced the anti-melanoma activity of a BRAF inhibitor, vemurafenib [86]. Similarly, α-humulene, which belongs to sesquiterpenoids intensified the anti-melanoma activity of trametinib, a MEK inhibitor [87]. Such synergistic treatment of natural compounds with drugs can result in lowering the drug dosage and alleviating the side effects of chemotherapy. Radiation is another commonly used therapy for skin cancers. However, the radiation resistance limits the effectiveness of this therapy. Some terpenes, including β-elemene and thymoquinone, are known to enhance the radiosensitivity of melanoma cells by inducing cell cycle arrest, apoptosis, and inhibiting DNA repair [88,89]. Although the synergistic effects of terpenoids discussed in this research (eucalyptol, eugenol, geraniol, linalool, and ursolic acid) have yet to be explored, we believe they could possibly have synergism since these terpenoids also induced cell cycle arrest and apoptosis. To enable their therapeutic application and boost their maximal anti-skin-cancer effects, such experiments should be continued.

## Figures and Tables

**Table 1:** Terpenoids against non-melanoma and melanoma skin cancer.

Terpenoids	Structure	Source	Effects and Mechanisms of Action	
Eucalyptol (Monoterpenoid)	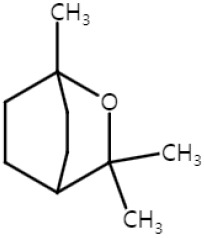	*Salvia fruticosa* *Eucalyptus globulus* *Rosmarinus officinalis*	Anti-non-melanoma effects	Mechanism of action
			anti-carcinogenesis pro-apoptotic anti-proliferation anti-metastasis	In vitro COX-2, PGE_2_ ↓ [31] G2/M cell cycle arrest, Bax/Bcl-2, Cyt-c, caspase 3, 9 ↑ [32], PI3K/Akt/mTOR ↓, vimentin, snail, slug, twist, MMP2, MMP9 ↓, n-cadherin ↓, E-cadherin ↑ [33]
			Anti-melanoma effects	Mechanism of action
			anti-metastasis	In vitro PI3K/Akt/mTOR ↓, vimentin, snail, slug, twist, MMP2, MMP9, n-cadherin ↓, E-cadherin ↑ [33] In vivo Vimentin ↓ [33]
Eugenol (Monoterpenoid)	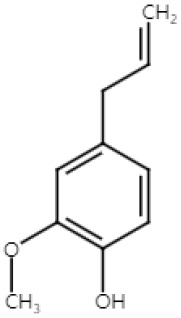	nutmeg cinnamon clove basil	Anti-non-melanoma effects	Mechanism of action
			anti-inflammation anti-proliferation pro-apoptotic	In vivo P53, P21^WAF1^ ↑, NF-κB, iNOS, COX-2, phospho-IkBα, IL-6, TNF-α, PGE2 ↓ [34] c-Myc, H-ras, Bcl-2 ↓, P53, Bax, caspase-3 ↑ [35]
			Anti-melanoma effects	Mechanism of action
			pro-apoptotic anti-proliferation anti-metastasis	In vitro E2F1 ↓, S-phase cell cycle arrest [36] In vivo Tumor growth delay, tumor size ↓ [36]
Geraniol (Monoterpenoid)	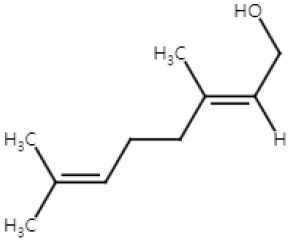	*Cinnamomum tenuipilum* *Phyla scaberrima* lemon grapefruit	Anti-non-melanoma effects	Mechanism of action
			pro-apoptotic anti-proliferation anti-metastasis	In vitro LOX-5, hyaluronidase ↓, G0/G1 cell cycle arrest [37] In vivo Edema, hyperplasia, COX-2, oxida-tive stress ↓ [38] Tumor incidence, number ↓ [38] RAS/Raf/ERK1/2 ↓, Bcl-2/Bax ↓ [38]
			Anti-melanoma effects	Mechanism of action
			pro-apoptotic	In vivo HMG CoA ↓ [39]
Linalool (Monoterpenoid)	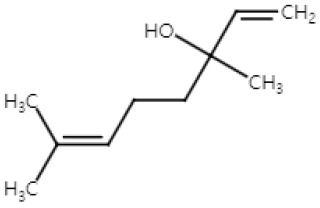	*Cinnamomum tenuipilum* *Coriandrum sativum* *Lavandula angustifolia*	Anti-non-melanoma effects	Mechanism of action
			anti-proliferation	In vivo NF-κB, TNF-α, IL-6 ↓ [40] COX-2, VEGF, TGF-β1, Bcl-2 ↓ [40]
			Anti-melanoma effects	Mechanism of action
			pro-apoptotic anti-proliferation anti-angiogenesis anti-metastasis	In vitro Caspase-3 ↑ [41] HIF-1α, VEGF ↓, vimentin, MMP2, MMP9 ↓ [42] E-cadherin ↑ [42]
Ursolic acid (Triterpenoid)	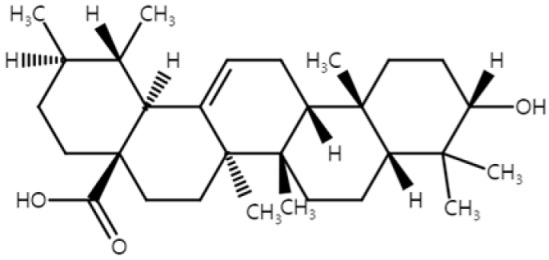	blueberry cranberry apple *Salvia rosmarinus*	Anti-non-melanoma effects	Mechanism of action
			pro-apoptotic anti-proliferation anti-neoplastic transformation	In vitro ROS ↓, caspase-3, -7 ↑ [43] Nrf2 ↑, HO-1, NQO1, UGT, GST ↑ [44] In vivo Nrf2, Nqo1 ↑ [45]
			Anti-melanoma effects	Mechanism of action
			pro-apoptotic anti-inflammation	In vitro P53, caspase-3 ↑, Bcl-2 ↓ [46] NF-κB, c-FOS, ATF-2, CREB-1 ↓ [46] TNF-α, IL-1β, IL-6, GM-CSF↓ [46] Caspase-3, -8 ↑, Bax ↑, Bcl ↓ [47,48] S-phase arrest [49]

↓ indicates a decrease in expression and ↑ indicates an increase in expression.

## Supplementary Materials

The supporting information can be downloaded at: https://www.mdpi.com/article/10.3390/ijms25084423/s1.
