# Supplementary material for: The Anticancer Activities of Natural Terpenoids That Inhibit Both Melanoma and Non-Melanoma Skin Cancers
Source: Int J Mol Sci. 2024 Apr 17;25(8):4423. doi: 10.3390/ijms25084423 (PMC11050645; doi:10.3390/ijms25084423)
Supplement: Supplementary file 1 [file ijms-25-04423-s001.zip › ijms-2889145-supplementary.pdf]

Supplementary material for:

Review

The Anticancer Activities of Natural Terpenoids That Inhibit Both Melanoma and Non-Melanoma Skin Cancers

Ye Eun Yoon [1,*], Young Jae Jung [1] and Sung-Joon Lee [2,3,*]

1 Department of Biotechnology, Graduate School of Life Sciences and Biotechnology, College of Life Sciences and Biotechnology, Korea University, Seoul 02855, Republic of Korea; lyjung51@korea.ac.kr
2 Department of Food Bioscience and Technology, College of Life Sciences and Biotechnology, Korea University, Seoul 02855, Republic of Korea
3 Interdisciplinary Program in Precision Public Health, BK21 Four Institute of Precision Public Health, Korea University, Seoul 02846, Republic of Korea
* Correspondence: yeeun916@korea.ac.kr (Y.E.Y.); junelee@korea.ac.kr (S.-J.L.)

Table S1. List of abbreviations

**AhR**	aryl hydrocarbon receptor
**ALT**	alanine aminotransferase
**AST**	aspartate aminotransferase
**BCC**	basal cell carcinoma
**CMM**	cutaneous malignant melanoma
**COX-2**	clyclooxygenase-2
**DMBA**	7, 12-dimethylbenz[a]anthracene
**EMT**	epithelial to mesenchymal transition
**5-FU**	5-fluorouracil
**GM-CSF**	granulocyte-macrophage colony-stimulating factor
**GST**	glutathione-S-transferase
**HMG-CoA**	3-hydroxy-3-methylglutaryl coenzyme A
**HO-1**	heme oxygenase-1
**LOX5**	lipoxygenase-5
**LVEF**	left ventricular ejection fraction
**NMSC**	non-melanoma skin cancer
**NQO1**	NAD(P)H:quinone oxidoreductase 1
**Nrf2**	nuclear factor E2-related factor 2
**PCNA**	proliferating cell nuclear antigen
**PGE2**	prostaglandin E2
**PKC**	protein kinase C
**ROS**	reactive oxygen species
**SCC**	squamous cell carcinoma
**TPA**	12-O-tetradecanoylphorbol-13-acetate
**UA**	ursolic acid
**UGT**	uridine 5′-diphospho-glucuronosyltransferase
**UV**	ultra violet
